# The role of horizontal transfer in the evolution of a highly variable lipopolysaccharide biosynthesis locus in xanthomonads that infect rice, citrus and crucifers

**DOI:** 10.1186/1471-2148-7-243

**Published:** 2007-12-06

**Authors:** Prabhu B Patil, Adam J Bogdanove, Ramesh V Sonti

**Affiliations:** 1 Centre for Cellular and Molecular Biology, Hyderabad-500007, India; 2 Department of Plant Pathology, Iowa State University, Ames, Iowa-50011, USA

## Abstract

**Background:**

Lipopolysaccharide (LPS) is a pathogen associated molecular pattern (PAMP) of animal and plant pathogenic bacteria. Variation at the interstrain level is common in LPS biosynthetic gene clusters of animal pathogenic bacteria. This variation has been proposed to play a role in evading the host immune system. Even though LPS is a modulator of plant defense responses, reports of interstrain variation in LPS gene clusters of plant pathogenic bacteria are rare.

**Results:**

In this study we report the complete sequence of a variant 19.9 kb LPS locus present in the BXO8 strain of *Xanthomonas oryzae *pv. *oryzae *(Xoo), the bacterial blight pathogen of rice. This region is completely different in size, number and organization of genes from the LPS locus present in most other strains of Xoo from India and Asia. Surprisingly, except for one ORF, all the other ORFs at the BXO8 LPS locus are orthologous to the genes present at this locus in a sequenced strain of *X. axonopodis *pv. *citri *(Xac; a pathogen of citrus plants). One end of the BXO8 LPS gene cluster, comprised of ten genes, is also present in the related rice pathogen, *X. oryzae *pv. *oryzicola *(Xoc). In Xoc, the remainder of the LPS gene cluster, consisting of seven genes, is novel and unrelated to LPS gene clusters of any of the sequenced xanthomonads. We also report substantial interstrain variation suggestive of very recent horizontal gene transfer (HGT) at the LPS biosynthetic locus of *Xanthomonas campestris *pv.* campestris *(Xcc), the black rot pathogen of crucifers.

**Conclusion:**

Our analyses indicate that HGT has altered the LPS locus during the evolution of *Xanthomonas oryzae *pathovars and suggest that the ancestor of all *Xanthomonas oryzae *pathovars had an Xac type of LPS gene cluster. Our finding of interstrain variation in two major xanthomonad pathogens infecting different hosts suggests that the LPS locus in plant pathogenic bacteria, as in animal pathogens, is under intense diversifying selection.

## Background

Lipopolysaccharide (LPS) is an essential outer membrane component of gram-negative bacteria. LPS is composed of Lipid A, core oligosaccharide and the O-antigen polysaccharide chain. LPS is highly immunogenic in animals with LPS recognition leading to induction of defense responses [1]. The loci that encode functions involved in LPS biosynthesis show enormous variation across different strains of the same species of animal pathogenic bacteria [2]. For example, eleven highly divergent gene clusters were reported to occupy an LPS locus in *Pseudomonas aeruginosa*, an opportunistic human pathogen [3]. This variation is attributed to selection to evade immune responses; in *Vibrio cholera *changes involving LPS loci within different strains have been implicated in major epidemics [4]. Apart from being a Pathogen Associated Molecular Pattern (PAMP), LPS also acts as a receptor for bacteriophages, with mutations that cause altered LPS resulting in phage resistance [5,6].

In plant pathogenic bacteria, LPS is an important virulence factor [6-12]. LPS is also being increasingly recognized as a major PAMP for plants [13-15] inducing expression of plant defense related genes, production of phenolic compounds and an oxidative burst, and suppressing hypersensitive reaction (HR) [14-18]. The induction of the nitric oxide synthase (NOS) gene is a hallmark of the mammalian immune response and recently, LPS has also been shown to induce the NOS gene in Arabidopsis [19]. Most of the above studies were carried out in dicot plants, but very recently LPS was also shown to induce defense responses and cell death in rice, a model monocot plant [20]. All of these findings clearly indicate an important role for LPS recognition in plant defense. Therefore, as in animal pathogenic bacteria, variation in LPS gene clusters might be expected across different strains of plant pathogenic bacteria.

*Xanthomonas *is a large genus of plant pathogenic bacteria that show a high degree of host plant and tissue specificity. One member of this genus, *X. oryzae *pv. *oryzae *(Xoo) causes bacterial blight, a serious disease of rice. LPS is an important virulence factor of Xoo [7] and in earlier work, we reported the complete sequence of an LPS gene cluster from our laboratory strain, BXO1 [21]. This gene cluster (henceforth referred to as the BXO1 type) was found in a majority of Xoo strains examined including two strains (Xoo KACC10331 and Xoo MAFF311018) that have been completely sequenced [22,23]. However, this gene cluster was absent in two strains of Xoo, BXO8 (a variant Indian pathotype) and Nepal624 [21]. In this paper, we report the complete sequence of the LPS gene cluster present in the BXO8 strain of Xoo. We compare this sequence with that of the LPS gene clusters of *X. axonopodis *pv. *citri *(Xac; a pathogen of citrus plants) and *X. oryzae *pv. *oryzicola *(Xoc; causal agent of bacterial leaf streak of rice and closely related to Xoo). We also report substantial interstrain variation at this locus among three strains of *Xanthomonas campestris *pv. *campestris *(Xcc), a destructive pathogen that causes black rot of cabbage and other cruciferous plants, including Arabidopsis. A meta-comparison across all examined variants of the LPS locus in different xanthomonads revealed relationships and an overall pattern that suggests multiple horizontal gene transfer (HGT) events and strong diversifying selection.

## Results

### Genetic organization of an LPS biosynthesis locus in the BXO8 strain of *Xanthomonas oryzae *pv. *oryzae*

**Figure 1:**
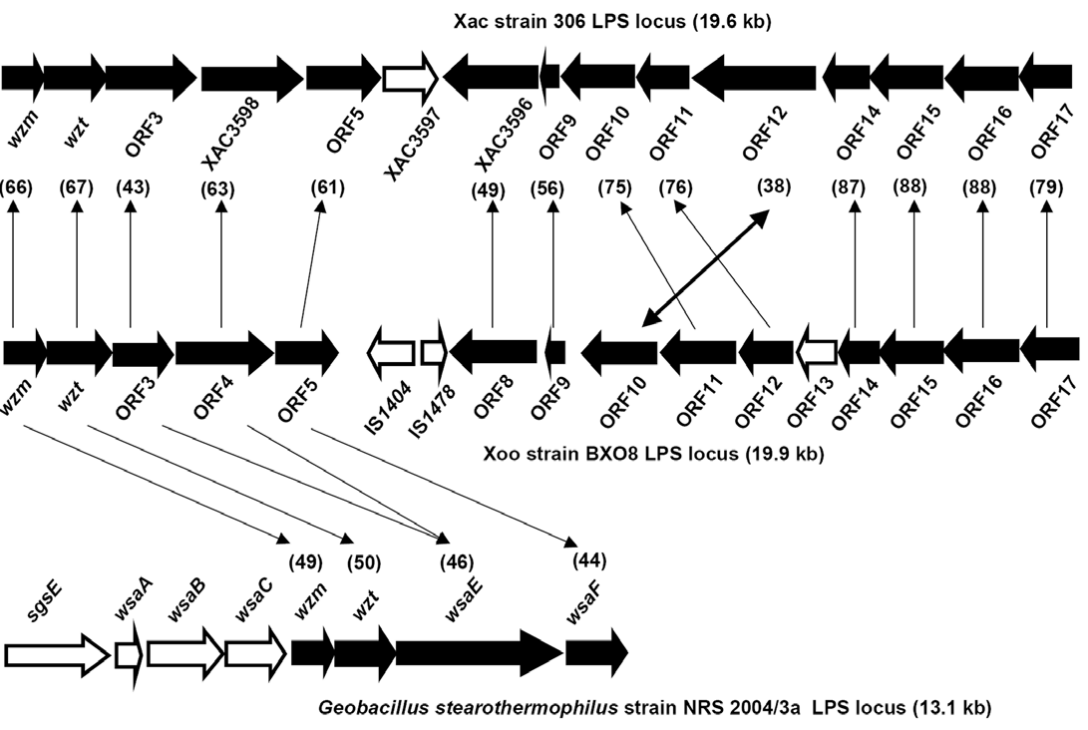
**Genetic organization of the LPS gene cluster of Xoo strain BXO8 and comparison to LPS gene clusters of Xac strain 306 and *Geobacillus stearothermophilus *strain NRS 2004/3a**. Orthologs are indicated by arrow lines. Rearrangement of ORF10 of BXO8 in comparison to its counterpart XAC3592 is indicated by a double headed arrow. ORFs shared by strains are represented by filled block arrows. ORFs specific to one of the gene clusters are represented by empty block arrows. Values in parentheses indicate pairwise % nucleotide identities of orthologs.

The sequence of the LPS biosynthetic gene cluster of BXO8 was obtained using long range PCR and shotgun sequencing of the PCR amplified product. The sequence was confirmed by resequencing on genomic DNA using PCR amplified products obtained with a set of nested primers that spanned the length of the LPS locus (see Methods). The locus is 19.9 kb and contains seventeen open reading frames (ORFs) (Fig. 1). As in Xoo strain BXO1 [21], the Xoo strain BXO8 LPS locus is flanked by the genes for cystathionine gamma lyase (*metB*) at one end and electron transport flavoprotein (*etfA*) at the other end. The size, number of genes encoded, and the organization of the BXO8 LPS locus are in contrast to the LPS locus that is present in Xoo strain BXO1 [21]. The BXO1 LPS gene cluster is > 99% identical to the 12.2 kb long, seven gene LPS gene cluster of Xoo strains KACC10331 [22] and MAFF311018 [23]. The overall G+C content of the Xoo strain BXO8 LPS locus is 56.7% (exclusive of IS elements), which is strikingly different from the average genomic G+C content of 63.7% for Xoo, based on the genome sequences of Xoo strains KACC10331 [22] and MAFF311018 [23]. However, there is variation in G+C content of individual ORFs of the BXO8 LPS gene cluster ranging from 46.8% for ORF9 to as high as 63% for ORF14.

The list of ORFs and their most similar homologs identified using BLASTX is given in Table 1. The first two ORFs from the *metB *side were annotated as putative ABC transporter permease (*wzm*) and putative ATP binding protein (*wzt*) genes. The start codon of *wzt *overlaps with the stop codon of *wzm*. The overlap between the start codon of *wzt *and the stop codon of *wzm *also suggests that these two genes are co-transcribed. Homologs of *wzt *and *wzm *are present in many LPS gene clusters. In BXO8, the *wzm *and *wzt *genes encode proteins of 260 and 410 amino acids respectively, that show significant similarity to the putative Wzm [AAM38444.1] and Wzt [AAM38443.1] proteins of Xac strain 306. ORF3 encodes a 386 aa protein of unknown function. The fourth ORF encodes a 611 aa protein that shows homology to a putative O-antigen biosynthesis protein encoding gene of Xac strain 306 [AAM38441.1]. However, BLAST search revealed that this gene is a truncated copy with most of the 5' region missing, when compared to its homologs from other bacteria (data not shown). Surprisingly all the homologous counterparts of ORFs 1 (*wzm*), 2 (*wzt*), 3, 4 and 5 exist in the same contiguous order and transcriptional direction in *Geobacillus stearothermophilus *strain NRS 2004/3a [Gst; AF328862] (Fig 1). In the BLASTX result, both ORF3 and ORF4 exhibit homology to different portions of the same protein [AAR99608] encoded by ORFG106 in Gst strain NRS 2004/3a, suggesting the possibility of a frameshift. ORF5, which encodes a hypothetical protein, is homologous to a glycosyl transferase in *Roseovarius *sp. 217 [ZP_01037878.1], *Acidothermus cellulolyticus *11B [ZP_01136681.1], and other bacteria.

**Table 1:** Homologs of predicted products of ORFs in the LPS locus of Xoo strain BXO8

**ORF (product size [aa])**	**Putative function**	**Homologous protein (size [aa])**	**Organism**	**Accession no.**	**I/S (E value)^*a*^**
ORF1 (260)	ABC-2 type transporter	ABC transporter permease (260)	Xac strain 306	AAM38444	70/84 (2e-85)
ORF2 (410)	ATP binding	ATP binding protein (409)	Xac strain 306	AAM38443	74/84 (4e-172)
ORF3 (386)	Unknown	Hypothetical protein (563)	Xac strain 306	AE008923	51/65 (5e-92)
ORF4 (611)	O-antigen biosynthesis	O-antigen biosynthesis protein (614)	Xac strain 306	AAM38441	66/79 (0)
ORF5 (417)	glycosyl transferase	Hypothetical protein (419)	Xac strain 306	AE008923	61/73 (5e-141)
ORF6 (311)	Transposase	IS*1404 *(349)	Xoo KACC10331	YP_202804	97/98 (4e-145)
ORF7 (158)	Transposase	IS*1478 *(158)	Xoo KACC10331	YP_201135	89/92 (1e-76)
ORF8 (521)	unknown	Hypothetical protein (581)	Xac strain 306	AAM38439	44/61 (3e-102)
ORF9 (136)	glycosyltransferase	Hypothetical protein (132)	Xac strain 306	AAM38438	50/70 (2e-33)
ORF10 (452)	Unknown	Hypothetical protein (746)	Xac strain 306	AAM38435	32/50 (7e-13)
ORF11 (430)	protoporphyrinogen oxidase	phytoene desaturase (428)	Xac strain 306	AAM38437	78/88 (0)
ORF12 (312)	epimerase	NAD dependent epimerase (314)	Xac strain 306	AAM38436	81/89 (4e-148)
ORF13 (223)	methyltransferase	Methyltransferase type 11 (229)	*Nocardioides *sp. JS614	ABL82494	48/64 (7e-52)
ORF14 (242)	dehydrogenase	Short chain dehydrogenase (242)	Xac strain 306	AAM38434	90/95 (3e-109)
ORF15 (433)	oxidoreductase	Putative oxidoreductase (433)	Xac strain 306	AAM38433	93/94 (0)
ORF16 (481)	prenyltransferase	Integral membrane protein (481)	Xac strain 306	AAM38432	95/97 (0)
ORF17 (328)	unknown function	Integral membrane protein (292)	Xac strain 306	AAM38431	90/93 (4e-126)

^*a *^I and S indicate identity and similarity, respectively. E values were obtained by using the BLASTX algorithm and screening the NCBI database.

The ORFs transcribed from the *etfA *side also exhibit interesting homologies. ORF16 and ORF17 encode 481 and 328 aa proteins respectively. Both show very high identity (> 90%; Table 1) to proteins [AAM38432.1 and AAM38431.1] annotated as putative integral membrane proteins in Xac strain 306. ORF15 encodes a 433 aa protein and also is homologous to a putative oxidoreductase of Xac strain 306 [AAM38433.1]. ORF14 encodes a 242 aa protein and is homologous with a putative shortchain dehydrogenase present in Xac strain 306 [AAM38434.1]. ORF13 encodes a 223 aa long protein homologous with a methyltransferase protein in *Nocardioides *sp. JS614 [ABL82494.1]. ORF12 encodes a 312 aa long protein homologous with a predicted NAD dependent epimerase in Xac strain 306 [AAM38436.1]. ORF11 is a 430 aa protein and shows homology to a putative phytoene desaturase of Xac strain 306 [AAM38437.1]. The next three ORFs (ORF8, ORF9 and ORF10) encode proteins of 521, 136 and 452 aa, respectively, annotated as putative transmembrane proteins in many bacteria. ORF9 shows significant homology to the glycosyltransferaseA (GtrA) family of proteins present in *Dechloromonas aromatica *(48% identity, 60% similarity), *Syntrophobacter fumaroxidans *(42% identity, 62% similarity), and other bacteria. Orthologs of ORF8, ORF9 and ORF10 are present in the LPS gene cluster of Xac strain 306 (Table 1).

### The Xoo strain BXO8 and Xac strain 306 LPS gene clusters are highly similar

The orthologs in Xac strain 306 of the BXO8 LPS biosynthetic genes mentioned above reside in a cluster between the conserved *metB *and *etfA *genes with overall high similarity to the BXO8 LPS cluster (Fig. 1). The size of the LPS locus is similar in both BXO8 (19.9 kb) and Xac strain 306 (19.6 kb). In both LPS gene clusters, the ORFs are organized in two convergent transcriptional units. We reannotated the Xac strain 306 LPS locus and identified two new ORFs, ORF3XAC and ORF5XAC. For all the ORFs of the BXO8 LPS locus, except for ORF13, orthologous counterparts exist in Xac strain 306. However, there is one minor rearrangement in the BXO8 LPS gene cluster. ORF10, the ortholog of XAC3592, is present between orthologs of XAC3595 and XAC3594, instead of after the ortholog of XAC3591. As in BXO8, the Xac LPS gene cluster encodes five ORFs (*wzm*, *wzt*, ORF3, XAC3598 and ORF5) homologous to the gene cluster of Gst strain NRS 2004/3a. The XAC3598 ORF is annotated as a truncated O-antigen biosynthesis protein gene, and both ORF3 and XAC3598 are homologous to different portions of the same ORF [AAR99608] in Gst strain NRS 2004/3a. Interestingly, there is no homolog of XAC3597 within either the LPS gene cluster of BXO8 or the Gst strain NRS 2004/3a locus.

BLAST comparisons of the BXO8 and Xac LPS loci using the web-based Artemis Comparison Tool revealed that nucleotide level identity varies for different genes and regions. The first region consisting of ORF1 to ORF5 of BXO8 exhibits around 63% nucleotide identity to the corresponding region of the Xac LPS locus. The second region comprising ORF8 to ORF10 is most diverged and has around 50% nucleotide identity. The third region comprising ORF11 and ORF12 shows around 70% nucleotide identity. The fourth region consisting of ORF14 to ORF17 is the least diverged and shows 89% identity at the nucleotide level. Pairwise comparison of the orthologous genes in the BXO8 and Xac LPS loci is shown in Fig. 1. The percentage of nucleotide identity varies from 38% (between ORF10 and XAC3592) to 88.2% (between ORF15 and XAC3591).

### The Xoc strain BLS 256 LPS gene cluster is chimeric

**Figure 2:**
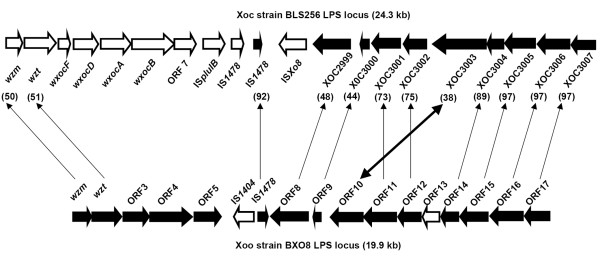
**Comparison of the LPS gene clusters of BXO8 and Xoc strain BLS256**. Orthologs are indicated by arrow lines. Rearrangement of ORF10 in comparison to its counterpart XOC3003 is indicated by a double sided arrow. ORFs shared by both strains are represented by filled block arrows. ORFs specific to one of the gene clusters are represented by empty block arrows. Values in parentheses indicate pairwise % nucleotide identities of orthologs. There is no ortholog of the BXO8 ORF13 in the Xoc strain BLS256 genome. There are no orthologs of BLS256 genes *wxocF*, *wxocD*, *wxocA*, *wxocB *and *orf7 *in the BXO8 LPS cluster.

The LPS biosynthetic locus present between *metB *and *etfA *in Xoc strain BLS 256 was retrieved from a whole, partially annotated, genome sequence available through the Comprehensive Microbial Resource [24]. The locus is 24 kb, bigger than the BXO1 (12.2 kb) and BXO8 (19.9 kb) LPS loci. The sequence of the Xoc LPS locus was annotated and compared with the corresponding region in BXO8 (Fig. 2). As in BXO8 and Xac, the genes in the LPS cluster appear to be organized in two blocks that are convergently transcribed. Interestingly, in the Xoc LPS locus, there are two full copies of the IS elements IS*plulB *and IS*1478 *at the junction of these convergently transcribed sets of genes. A copy of the IS element IS*1478 *is also present in the BXO8 LPS region at the same position (Fig. 2). Moreover, the block of (nine) genes at the *etfA *end is orthologous with the Xac strain 306 and BXO8 clusters. However, unlike in BXO8, there is no rearrangement of the homolog of XAC3592. BLASTN comparison of the LPS locus of BXO8 to those of Xac strain 306 and Xoc strain BLS256 was done using Artemis Comparison Tool, and the region comprising ORF14 to ORF17 of BXO8 was found to exhibit more identity to Xoc strain BLS256 (96%) than to Xac strain 306 (89%).

There is however, very little similarity in the block of genes that begin from the *metB *side, except for *wzm *(50% nucleotide identity) and *wzt *(51% identity). The *wzm *ORF encodes a 280 aa size protein that shows homology to a putative transporter permease [ABB75686] of *Nitrosospira multiformis *ATCC 25196 (61% identity and 75% similarity). The *wzt *ORF encodes a 452 aa protein that shows homology to a putative ATP binding protein [ABE29200] of *Burkholderia xenovorans *LB400 (56% identity and 74% similarity). The *wxocF *ORF encodes a 211 aa protein that shows homology to a putative acetyltransferase [AAY95934] of *Pseudomonas fluorescens *Pf-5 (48% identity and 63% similarity). The *wxocD *ORF encodes a 211 aa protein that is homologous to a glycosyltransferase [EAN25861] of *Pelodictyon phaeoclathratiforme *BU-1 (53% identity and 69% similarity). The *wxocA *ORF encodes a 438 aa protein that shows homology to a Lipopolysaccharide biosynthesis protein-like protein [EDK72384] of an uncultured bacterium belonging to candidate division TM7 genomosp. GTL1 (62% identity and 74% similarity). The *wxocB *ORF encodes a 596 aa protein that is homologous to a Lipopolysaccharide biosynthesis protein-like protein [EDK72384] of the uncultured bacterium candidate division TM7 genomosp. GTL1 (31% identity and 49% similarity). ORF 7 encodes a 280 aa protein that shows homology to a conserved hypothetical protein [EAR73134] of *Bacillus weihenstephane *KBAB4 (36% identity and 54% similarity). Overall, Xoc strain BLS 256 has a hybrid LPS gene cluster, with one part similar to BXO8 and Xac strain 306, and the other half being very different.

### Comparison of the LPS gene clusters of Xoo strain BXO8 and Xac strain 306 to *Stenotrophomonas maltophilia *strain R551-3

**Figure 3:**
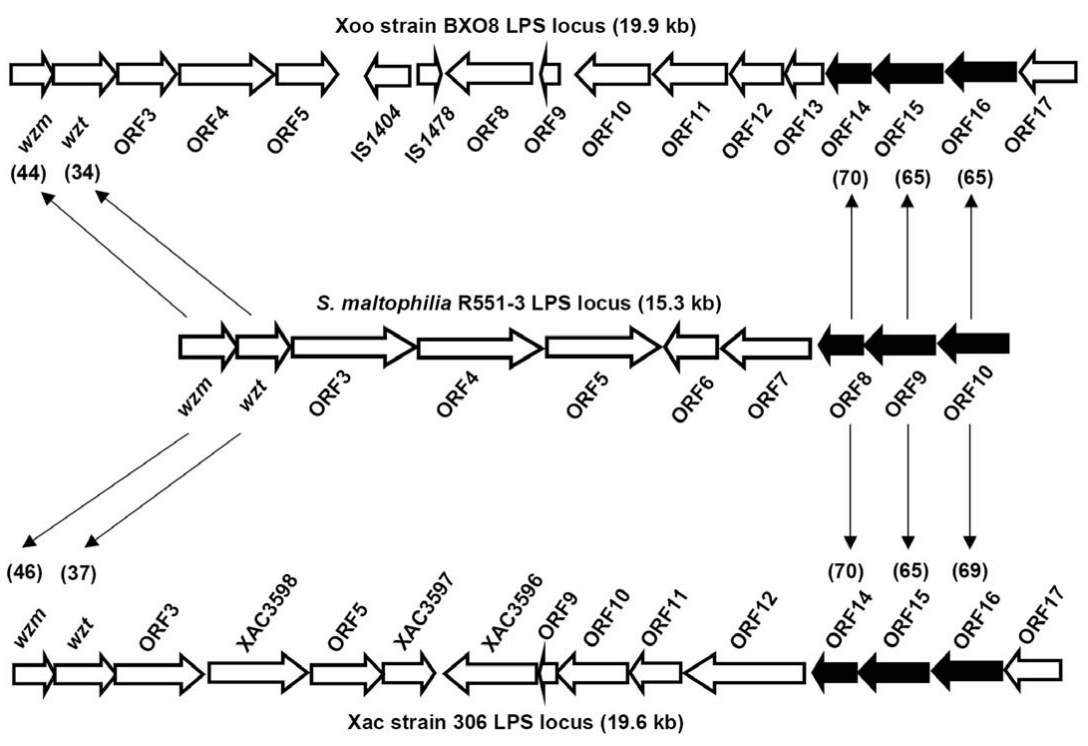
**Comparison of the LPS gene clusters of Sma strain R551-3 with Xoo strain BXO8 and Xac strain 306**. Orthologs are indicated by arrow lines. ORFs common to all three strains are represented by filled block arrows and ORFs not common to all three strains are represented by empty arrows. Values in parentheses indicate pairwise % nucleotide identities of orthologs.

*Stenotrophomonas maltophilia *belongs to the Xanthomonadaceae but is not phytopathogenic. Whole genome shotgun sequence of a *Stenotrophomonas maltophilia *(Sma) strain R551-3 is available through NCBI [NZ_AAVZ00000000]. Interestingly, a 15.3 kb gene cluster that encodes 10 ORFs, several of which have predicted functions involved in LPS biosynthesis, is located between *metB *and *etfA *[AAVZ01000014]. The organization of these 10 ORFs is shown in Fig 3 and the list of ORFs and their predicted functions is given in additional file 1. The first two ORFs from the *metB *side encode a putative ABC transporter permease (*wzm*) and putative ATP binding protein (*wzt*) genes that are present in many LPS gene clusters. The *wzm *and *wzt *genes of Sma R551-3 strain exhibit 44% and 34% nucleotide sequence identity to their BXO8 orthologs and 46% and 37% identity to their counterparts in Xac strain 306. The next five genes (ORFs 3 – 7) in the LPS gene cluster of Sma strain R551-3 are unrelated to genes in the LPS loci of Xoo strain BXO8 and Xac strain 306. Strikingly, the last three ORFs in the LPS gene cluster of Sma strain R551-3 are orthologous to predicted short chain dehydrogenase, oxidoreductase and integral membrane protein encoding genes that are located near the *etfA *end of the LPS gene clusters of BXO8, Xac strain 306 and Xoc strain BLS256. A nucleotide level comparison of these genes in the LPS gene cluster of Sma strain R55C-3 to those of their orthologs in Xoo strain BXO8 and Xac strain 306 strain is shown in Fig 3.

### Comparison of the LPS gene clusters of three Xcc strains and *Xanthomonas campestris *pv. *armoraciae *strain 756C

**Figure 4:**
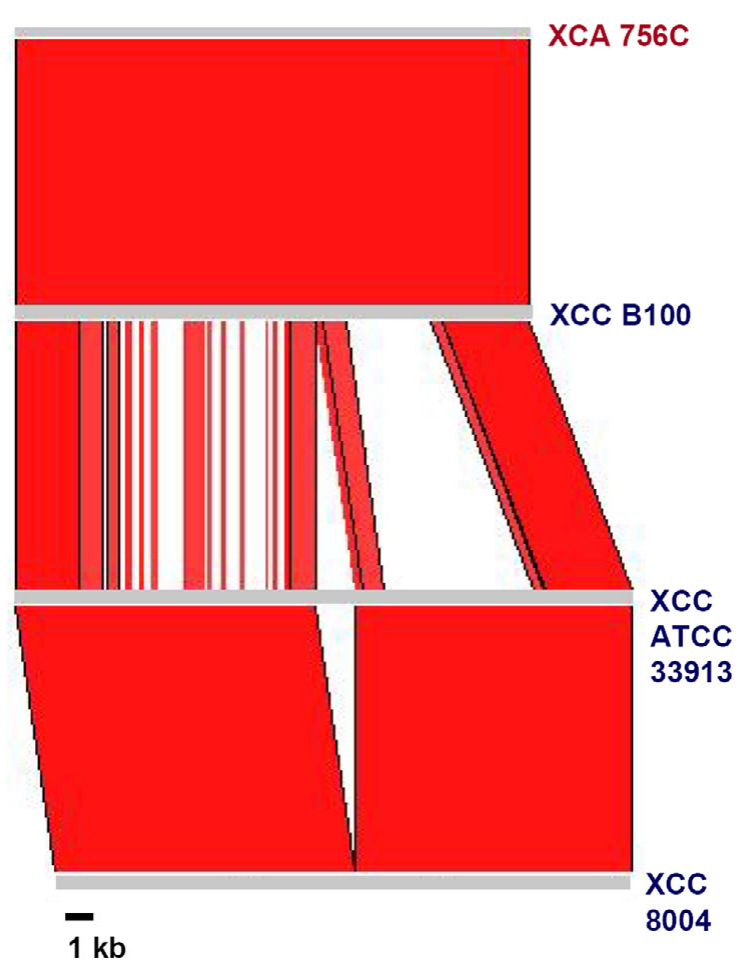
**Nucleotide level comparison of LPS gene clusters of three strains of Xcc and one strain of Xca**. Grey bars represent the LPS loci of the strains, as indicated. Comparisons were carried out using the BLASTN option of Artemis Comparison Tool (ACT). Bright red colour indicates a high level of nucleotide identity (95 to 100%) and gaps indicate highly diverged regions.

Nucleotide level comparison of LPS gene clusters of three Xcc strains, ATCC33913 [25], 8004 [26], and B100 [27], is shown in Fig. 4. The sizes of the LPS loci of these strains are 21.2 kb 19.9 kb, and 17.7 kb, respectively. One reason for the differences in size is the presence of IS elements in ATCC33913 (four insertions) and in 8004 (three insertions). The G+C content of each locus is around 58%. The LPS gene clusters of ATCC33913 and 8004 exhibit almost 100% nucleotide identity. Surprisingly, the LPS locus of B100 exhibits only 69% nucleotide identity to the LPS locus of these two strains. Only regions of 2 kb and 3 kb at the two extremities of the LPS locus of B100 exhibit high nucleotide identity (around 95%) with the LPS gene clusters of ATCC33913 and 8004.

TBLASTX comparisons of the LPS loci of these three Xcc strains identified one region that is specific to strain B100. This region is annotated to contain a *wxcO *gene encoding a putative integral membrane protein of 750 amino acid length [27]. In the other two Xcc strains, this region is annotated to contain an unknown gene (XCC0613 in ATCC33913 and XC_3621 in 8004) encoding a hypothetical protein of 730 amino acids. Interestingly, a homolog of *wxcO *is present in strain 85-10 of *X. axonopodis *pv. *vesicatoria *{Xav, a pathogen of tomato and pepper plants; formerly *X. campestris *pv *vesicatoria *[28]}, where it is annotated as a putative carbohydrate translocase (YP_365447.1; identity 76% and similarity 87%) [29] and in *Yersinia bercovieri *ATCC43970, where it is annotated as a hypothetical protein [ZP_00820359.1; identity 36% and similarity 54%). Homologs of XCC0613 and XC_3621 were not identifiable through BLASTX in any other bacteria. The XCC0613/XC_3621 ORF has a G+C content of 53.5%, which is substantially lower than average for other xanthomonad genes, and is suggestive of its acquisition by HGT. The fact that this ORF is not present in the LPS clusters of Xcc strain B100 and Xav strain 85-10, and that a different ORF (*wxcO*) is present at the corresponding position in these bacteria, is an additional indication that this ORF might have been acquired by HGT.

*X. campestris *pv. *armoraciae *(Xca) is the causal agent of bacterial leaf spot of cruciferous plants and is closely related to Xcc. As indicated in Table 2, the genome sequence of Xca strain 756C is available through the Comprehensive Microbial Resource [24]. The results of nucleotide level comparison of the LPS gene clusters of Xcc strains ATCC339193, 8004, and B100 with Xca strain 756C is shown in Fig. 4. The LPS gene cluster of B100 exhibits almost 100% nucleotide sequence identity to the LPS gene cluster of Xca strain 756C. The size of the LPS gene cluster is 17.7 kb in both strains.

**Table 2:** Sources of the sequences of LPS gene clusters compared in the present study

**Name of the strain/pathovar**	**Source**	**Accession or version number**
*Xanthomonas oryzae *pv. *oryzae *strain BXO1	NCBI	AF337647
*Xanthomonas oryzae *pv. *oryzicola *strain BLS256	TIGR	Ver02 (Comprehensive Microbial Resource) CMR release Mar 06
*Xanthomonas axonopodis *pv. *citri *strain 306	NCBI	NC_003919
*X. campestris *pv. *campestris *strain ATCC 33913	NCBI	NC_003902
*X. campestris *pv. *campestris *strain 8004	NCBI	NC_007086
*X. campestris *pv. *campestris *strain B100	NCBI	AF204145
*X. campestris *pv. *armoraciae *strain 756C	TIGR	Ver19, CMR April 18, 2006
*X. axonopodis *pv. *vesicatoria *strain 85-10	NCBI	NC_007508
*Stenotrophomonas maltophilia *strain R551-3	NCBI	AAVZ01000014

**Figure 5:**
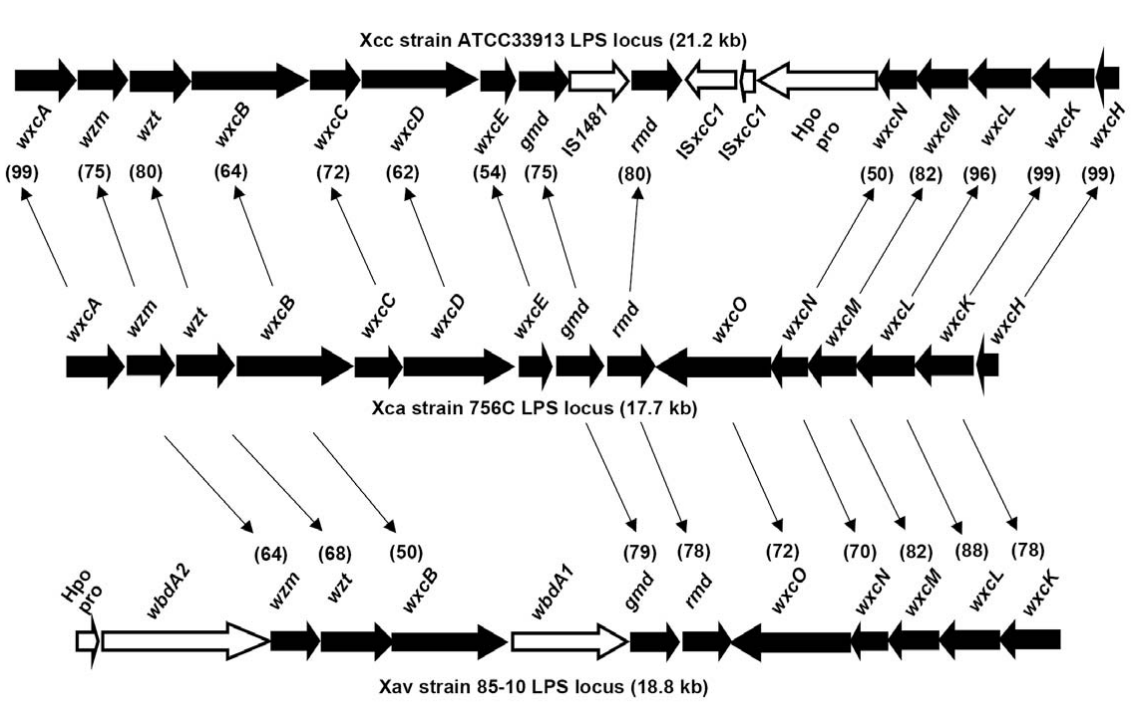
**Comparison of the LPS gene clusters of Xca strain 756C with Xcc strain ATCC33913 and Xav strain 85-10**. Orthologs are indicated by arrow lines. ORFs common to all three strains are represented by filled block arrows and ORFs not common to all three strains are represented by empty arrows. Values in parentheses indicate pairwise % nucleotide identities of orthologs. Hpo pro indicates a hypothetical protein encoding ORF.

A comparison of the LPS locus of Xca strain 756C with that of Xcc strain ATCC33913 and Xav strain 85-10 is provided in Fig 5. Except for *wxoO*, all of the other 14 ORFs of the LPS gene cluster of Xca strain 756C have orthologs in the LPS locus of Xcc strain ATCC33913. Four of these orthologs, *wxcA, wxcL, wxcK *and *wxcH*, have a very high level of nucleotide sequence identity (> 96%). The remaining 10 orthologs exhibit a significantly lower level of sequence identity (Fig. 5). The LPS gene cluster of Xav strain 85-10 contains orthologs of ten of the genes in the LPS gene cluster of Xca strain 756C with varying degrees of nucleotide sequence identity. The LPS gene cluster of Xav strain 85-10 does not contain orthologs of *wxcA, wxcC, wxcD, wxcE and wxcH*. However, the Xav strain 85-10 LPS locus has three unique ORFs which are *wxdA2, wxdA1 *and an ORF that encodes a hypothetical protein (Fig. 5).

## Discussion

Extensive interstrain differences are observed among LPS biosynthetic gene clusters of animal pathogenic bacteria [2]. These variations reflect the highly immunogenic nature of LPS and a necessity for the pathogen to vary LPS to evade detection by the host. Studies relating to LPS in phytopathogenic bacteria mostly describe either effects on virulence of mutations in LPS biosynthetic gene clusters or the role of LPS in inducing defense responses. The present study extends our previous work demonstrating interstrain variations at LPS loci in plant pathogenic bacteria and provides important insights into the extent of variation and the evolution of LPS gene clusters in different Xanthomonads. We report that the BXO1 and BXO8 strains of Xoo have LPS gene clusters that are completely different in sequence, number and organization of genes. The 12.2 kb LPS gene cluster of BXO1, which is also present in Xoo strains KACC10331 and MAFF311018, does not show significant similarity to the LPS gene clusters of any of the other sequenced xanthomonads, while the BXO8 LPS gene cluster is highly similar to the LPS locus of Xac strain 306, a citrus pathogen. The BXO1 strain belongs to pathotype 1b [30] of Xoo in India while the BXO8 strain represents a different pathotype, not yet given a designation but similar to pathotype 2 [30,31]. It remains to be determined whether the differences in pathological properties between the BXO1 and BXO8 strains depend on the differences in their LPS gene clusters.

Interestingly, in strain BLS256 of the related xanthomonad, Xoc, only one half of the LPS gene cluster is similar to that of the LPS loci of BXO8 and Xac strain 306. This suggests the possibility that the ancestor of both *X. oryzae *pathovars had an Xac strain 306 type of LPS gene cluster, that one HGT event in the ancestor of the Xoc lineage replaced part of a Xac strain 306 type of gene cluster with novel sequences, and that one more HGT event occurred to introduce a totally new LPS gene cluster into the ancestor of most Xoo strains. In previous work, we showed that the BXO1 type of LPS cluster is present in Xoo strains from India, Nepal, China, Malaysia, Indonesia, the Philippines and Korea [21]. This suggests that the HGT event which led to the introduction of the BXO1 type of LPS cluster into the Xoo genome must have occurred before the dispersal of the ancestor of these strains to the many widely separated locations in which they are found.

Genes at the LPS loci are important contributors to pathogenesis. Mutations at the LPS loci in Xoo and Xcc result in severe virulence deficiency [7,8,11,26]. A mutation in the putative *wzt *gene of BXO8 results in a LPS defect and severe virulence deficiency (PBP and RVS, unpublished data). A mutation in the *wxocB *gene in the LPS locus of Xoc strain BLS256 also reduces virulence (L. Wang and AJB, unpublished data). These results when taken together indicate that, in spite of largescale variations, the LPS locus likely plays a central role in pathogenesis in most xanthomonads that infect plants. One striking finding of the BLAST searches was that the first five ORFs (*wzm, wzt*, ORF3, ORF4 and ORF5) of the BXO8 and Xac strain 306 LPS loci have homologs in Gst strain NRS 2004/3a. Gst is a gram-positive thermophile and these genes are predicted to be involved in glycosylation of a surface (S) layer protein [32]. It is possible that a metagenome repertoire is contributing, to disparate bacteria, genes that participate in LPS biosynthesis and/or S-layer glycosylation.

The sequences of LPS gene clusters from three Xcc strains also gave evidence of HGT. The LPS gene cluster of Xcc strain B100 is significantly different from the LPS cluster present in two other strains of Xcc. Surprisingly, the nucleotide sequence of the Xcc B100 LPS locus is almost 100% identical to the LPS gene cluster of Xca strain 756C. One possibility is that the LPS gene cluster of Xcc strain B100 was acquired by a HGT event from an Xca strain. Since Xcc and Xca infect the same hosts, there is opportunity for such a genetic exchange to occur. The high level of nucleotide sequence similarity (almost 100%) between the LPS gene clusters of Xcc strain B100 and Xca strain 756C suggests that, if such a genetic exchange did occur, it must have happened fairly recently. Alternatively, Xca may have diverged recently from an Xcc strain with a B100 type LPS locus. The frequency with which the two divergent LPS gene clusters are present in different Xcc and Xca strains (world wide) remains to be determined and would help to clarify the likely scenario.

**Figure 6:**
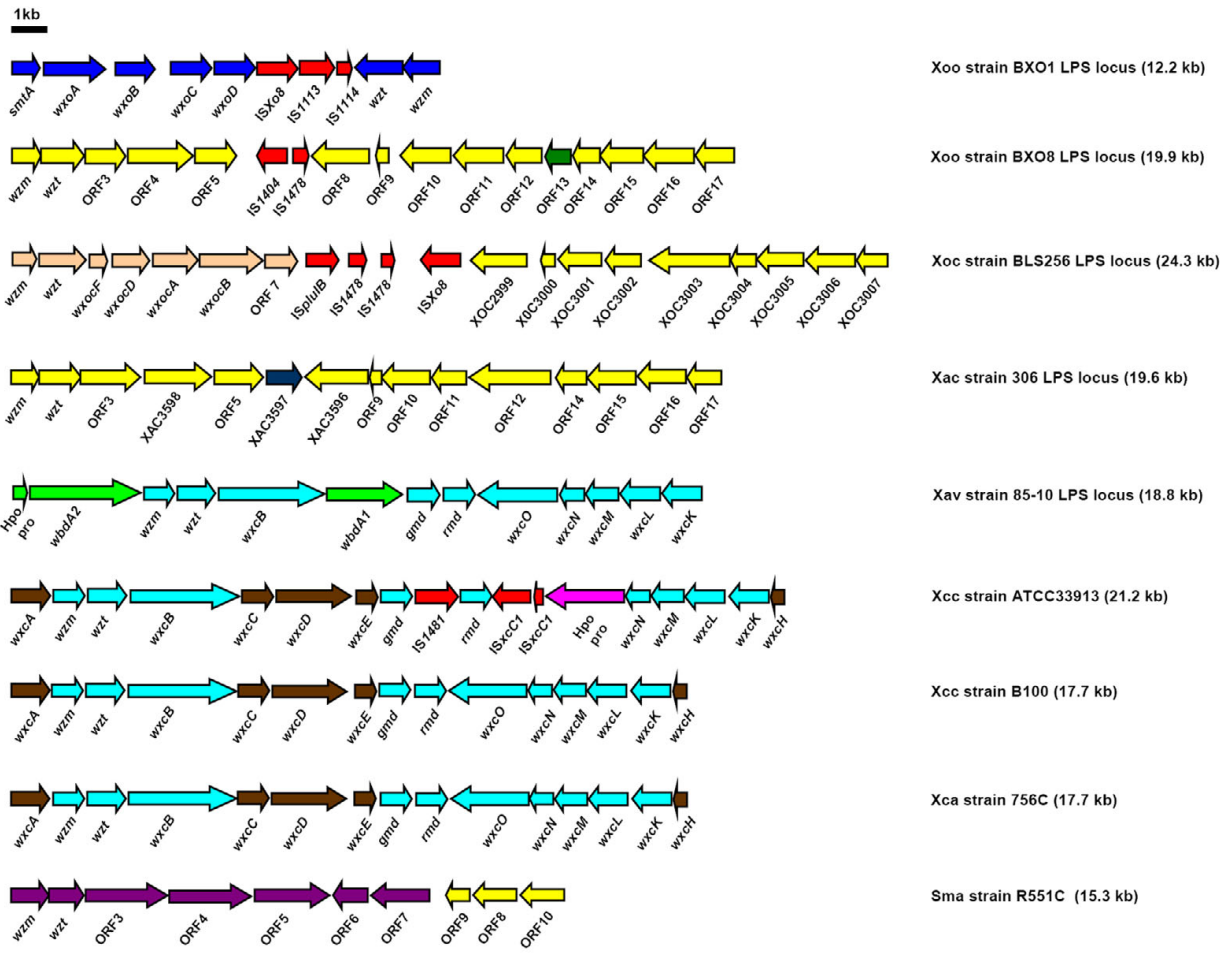
**A schematic comparison of LPS gene clusters described in the present study**. Genes conserved in different strains are given identical colour. Genes specific to individual strains are given unique colour. ORFs marked by red colour represent *IS *elements. Hpo pro indicates a hypothetical protein encoding ORF.

**Figure 7:**
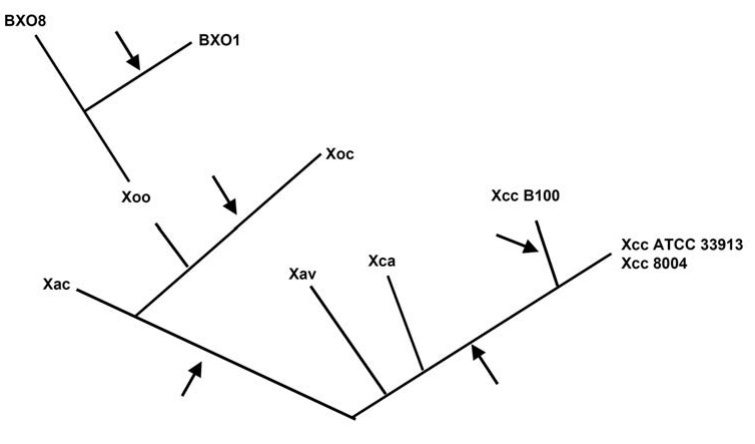
**Deduced pattern of horizontal gene transfer at an LPS locus in Xanthomonas species**. A hypothetical tree depicting putative horizontal gene transfer events (indicated by arrows) that gave rise to LPS gene clusters in different *Xanthomonas *species and pathovars.

In the genomes of all sequenced xanthomonads, there is an LPS locus between the *metB *and *etfA *genes. However, what is present at this locus is variable. A schematic diagram of the different xanthomonad LPS gene clusters compared in this study is shown in Fig. 6. It appears that multiple horizontal gene transfer events in the Xanthomonas lineage have contributed to this diversity. The genome of the pepper and tomato pathogen Xav strain 85-10 is more related to that of Xac strain 306 than either is to Xcc [29]. Even though both the *Xanthomonas axonopodis *pathovars are closely related, Xac strain 306 and Xav strain 85-10 have entirely different LPS gene clusters, the LPS locus of Xav strain 85-10 being similar, in overall gene organization and content, to that of Xcc strain B100 and Xca 756C. This suggests that at least one HGT has occurred in the ancestor of Xac and Xav. A 2.1 kb ORF (XCC0613/XC_3621O) in Xcc strains ATCC33913 and 8004 encodes a hypothetical protein that has no orthologs in the sequenced xanthomonads. We propose that a HGT event might have introduced this unique ORF into the genome of the ancestor of Xcc strains ATCC33913 and 8004. Overall, the results of this study indicate that multiple HGT events have occurred at this locus in the xanthomonads and that the variation introduced by HGT can involve either the entire LPS locus or part of the locus. A hypothetical evolutionary tree illustrating a pattern of HGT events that could explain the relationships observed among the LPS gene clusters of the *Xanthomonas *strains examined here is presented in Figure 7.

Our finding of large scale interstrain variation mediated by HGT at an LPS locus, in two important xanthomonad pathogens infecting diverse hosts, suggests that plant pathogenic bacteria are under selection to vary their LPS gene clusters i.e., that variations have an important adaptive value. LPS is known to strongly modulate plant immune responses [14]. The study of Desaki et al [20] indicates that the intensity of defense responses of rice cells towards LPS from pathogens and non-pathogens is different and suggests that recognition of LPS is important. Gross et al [33] suggest that Xcc LPS is taken up into tobacco cells through receptor mediated endocytosis. Variation in LPS biosynthetic gene clusters may block recognition/uptake and help in evading the innate immune responses of the host. An alternative or additional possibility is that variability is driven by selection for resistance to bacteriophages that infect xanthomonads.

*Stenotrophomonas maltophilia *belongs to the Xanthomonadaceae family. It is found in a wide range of environmental habitats, including agricultural settings, and is also an important cause of nosocomial infections. The genome sequence of *Stenotrophomonas maltophilia *strain R551-3, which is an endophyte of poplar [34], is available at NCBI. Interestingly, as in the *Xanthomonas *strains, in *Stenotrophomonas maltophilia *an LPS gene cluster is present between *metB *and *etfA*. In addition, it is pertinent to note that some of the genes in this locus are also related to genes in the LPS clusters of the Xoo BXO8 and Xac 306 strains.

A distinct locus also required for LPS biosynthesis is present near the *etfA/metB *locus in Xcc strain B100 and consists of *rmlA, rmlB, rmlC, rmlD *(required for synthesis of dTDP-Rhamnose), *xanA, xanB *(involved in UDP-Glucose and GDP-Mannose biosynthesis) and *lpsI, lpsJ *(3 Oxoacid CoA transferases) [35-37]. All of these genes are highly conserved in each of the other sequenced xanthomonads (more than 80% nucleotide identity) and in *Stenotrophomonas maltophilia *(more that 75% nucleotide identity) [[38]; data not shown]. As indicated above, these genes are involved in the synthesis of LPS precursors. The lack of variation in these genes suggests that these LPS precursors are conserved constituents of LPS in all xanthomonads.

Pathogenic members of the genus *Xanthomonas *cause diseases on at least 390 plant species. Currently, we have knowledge of the LPS gene clusters representing only a handful of *Xanthomonas *species and pathovars. Analysis of the LPS biosynthetic loci of a larger set of strains would shed further light on the extent of variation in LPS gene clusters in the xanthomonads and on the evolution that has been occurring at this locus, as well as the selective forces driving that evolution. As indicated in this study, long range PCR can be used effectively to isolate and sequence the LPS locus in diverse xanthomonads using primers that are targeted to the conserved *metB *and *etfA *genes.

## Conclusion

The LPS locus in the BXO8 strain of the rice pathogen Xoo is orthologous to the LPS locus of Xac, a xanthomonad that infects citrus. The LPS locus in another *X. oryzae *pathovar that infects rice, Xoc, is chimeric, with one half of this locus being orthologous to the LPS locus in BXO8 and Xac. A comparison of these LPS loci indicates that the ancestor of all *X. oryzae *pathovars is likely to have had an LPS locus that is related to the corresponding locus in Xac with variations in the *X. oryzae *lineage being introduced by several largescale HGT events. This study also presents interstrain variation at the LPS locus that provides evidence of HGT in Xcc, a xanthomonad pathogen that infects crucifer plants, indicating that interstrain variation in LPS biosynthetic genes is not a phenomenon restricted to *X. oryzae *pathovars. Overall, the pronounced variation in LPS biosynthetic gene cluster content across the several *Xanthomonas *strains described here suggests diversifying selection consistent with LPS playing a pivotal role in the interactions of members of this important group of plant pathogens with their hosts. Further, the analysis reveals that HGT has played a major part in generating this variation.

## Methods

### Bacterial strains and media

*Xanthomonas oryzae *pv. *oryzae *strains were grown at 28°C in Peptone Sucrose (PS) medium [39]. *E. coli *strains were grown at 37°C in Luria-Bertani (LB) medium [40]. Ampicillin was used at 100 μg ml^-1^.

### Long Range PCR

The BXO8 LPS locus was amplified using long range PCR (Triple Master™, Eppendorf, Hamburg, Germany) with genomic DNA as template per manufacturer's instructions. The primers BPMR1 and BPMR2 were used (Additional file 2). These primers were designed using previously available sequences from the junctions of the *metB *and *etfA *genes with the LPS locus [21]. An annealing temperature of 60°C and annealing time of 30 seconds were used for each cycle. Extension time was 24 minutes for each cycle. A total of 35 cycles were run. After the first ten cycles, extension time was increased by 10 seconds each cycle. An 18 kb PCR product was obtained using an extension time of 24 minutes for each cycle but not using 20 minutes extension time (Additional file 3). As expected, a similar size PCR product was also obtained using genomic DNA of Nepal624 as a template. The PCR product was gel eluted and end sequenced to confirm the authenticity of the region amplified.

### Shotgun sequencing

For shotgun sequencing, the gel eluted long range PCR product was partially digested using *Hae*III (New England Biolabs [NEB], Beverly, MA). These fragments were subsequently cloned in pMOS vector (Amersham Pharmacia Biotech, Buckinghamshire, England). Recombinant colonies were screened for the presence of insert using vector primers M13F (TGTAAAACGACGGCCAGT) and T7 (TAATACGACTCACTATAGGG). Only those colonies, having insert size between 1 kb to 3 kb were selected. Plasmids were isolated by using a modified alkaline lysis protocol [41], and inserts were sequenced using vector specific primers (M13F and T7) on an ABI Prism 3700 automated DNA sequencer (Applied Biosystems, Foster City, CA). After editing, the sequence data were assembled using GeneTools (BioTools, Alberta, Canada) and BLAST2 [42]. Multiple single strand sequences (3–8 X coverage) were generated for each region in the sequence. Gaps in the contig were filled and particular regions were confirmed by designing new primers (Additional file 2). For further confirmation, new overlapping primers at every 1 kb of the BXO8 LPS locus (Additional file 4) were used to reamplify and resequence the corresponding regions from BXO8 genomic DNA. The final reconfirmed and resequenced region of the BXO8 LPS locus was annotated using NCBI ORF finder [43].

### Comparison of LPS gene clusters

The complete sequences of the LPS gene clusters present in different *Xanthomonas *species and pathovars were retrieved from whole genome sequences, available through NCBI or TIGR websites (Table 2) except for the sequence of the cluster in B100, which was previously deposited in the NCBI database under the accession number AF204145[27], and that of Xoo strain BXO1 which was previously deposited in the NCBI database under the accession number AF337647[21]. The LPS loci of Xac and Xoc were reannotated using ORF finder through NCBI [43]. Homology searches were done using BLAST [44] and FASTA [45]. Nucleotide level identity of orthologous genes was estimated using the program needle of the EMBOSS package [46]. Overall comparison of different LPS loci was done using the web based Artemis Comparison Tool [47].

### Nucleotide sequence accession number

The 20070-bp sequence of BXO8 LPS locus described in the present study has been deposited in GenBank under the accession number DQ907230

## Authors' contributions

PBP performed the cloning, sequencing, and comparative analyses, and drafted the manuscript. AJB assisted in the analysis of the Xoc and Xca gene clusters and contributed to preparation of the final manuscript. RVS conceived and coordinated the study, and edited the final manuscript. All authors have read and approved the paper.

## Supplementary Material

Additional file 1 Homologs of predicted products of ORFs in the LPS locus of *Stenotrophomonas maltophilia *strain R551-3. Click here for file

Additional file 2 List of primers used in this study. Click here for file

Additional file 3 **Amplification of the BXO8 LPS gene cluster using long range PCR**. (A) The results of long range PCR using different extension times. A high molecular weight PCR product can be seen only after using an extension time of 24 minutes (see methods). M is the lamda *Hind*III marker. (B) The size of the PCR product was estimated in both BXO8 (lane2) and Nepal624 (lane3) to be around 18 kb. M1 and M2 are lambda *Hind*III and lamda monocut mix markers, respectively. Click here for file

Additional file 4 List of primers used for resequencing of the BXO8 LPS locus. Click here for file
