# Supplementary material for: The role of horizontal transfer in the evolution of a highly variable lipopolysaccharide biosynthesis locus in xanthomonads that infect rice, citrus and crucifers
Source: BMC Evol Biol. 2007 Dec 6;7:243. doi: 10.1186/1471-2148-7-243 (PMC2238763; doi:10.1186/1471-2148-7-243)
Supplement: Additional file 1 — Homologs of predicted products of ORFs in the LPS locus of Stenotrophomonas maltophilia strain R551-3. [file 1471-2148-7-243-S1.doc]

**Additional file 1. Homologs of predicted products of ORFs in the LPS locus of *Stenotrophomonas maltophilia* strain R551-3**

| **ORF (product size [aa])** | **Predicted function** | **Homologous protein (size [aa])** | **Organism** | **Accession no.** | **I/S (E value)*^a^*** |
| --- | --- | --- | --- | --- | --- |
| ORF1 (287) | ABC-2 type transporter | ABC transporter permease (285) | *Azospirillum brasilense* | AAS83021 | 40/66  (1e-52) |
| ORF2 (242) | ATP binding | ATP binding protein (242) | *Thiobacillus denitrificans* ATCC 25259 | AAZ97830 | 51/73  (7e-67) |
| ORF3 (747) | unknown | hypothetical protein (724) | *Myxococcus xanthus* DK 1622 | ABF89742 | 38/52  (7e-73) |
| ORF4 (724) | glycosyltransferase | Glycosyltransferase  (780) | *Myxococcus xanthus* DK 1622 | ABF90679 | 32/49  (1e-71) |
| ORF5 (675) | glycosyltransferase | Glycosyltransferase  (780) | Myxococcus xanthus  DK 1622 | ABF90679 | 27/42  (3e-30) |
| ORF6 (337) | epimerase | UDP-glucose 4-epimerase (336) | *Delftia acidovorans* SPH-1 | EAV76740 | 57/71  (7e-107) |
| ORF7 (533) | unknown | unknown protein | no significant homolog | Not Applicable | Not applicable |
| ORF8 (244) | dehydrogenase | Short chain dehydrogenase  (242) | Xac strain 306 | AAM38434 | 74/84  (1e-83) |
| ORF9 (438) | oxidoreductase | Oxidoreductase  (433) | Xac strain 306 | AAM38433 | 67/75  (2e-156) |
| ORF10 (473) | prenyltransferase | Integral membrane protein (481) | Xac strain 306 | AAM38432 | 73/84  (4e-158) |

*^a^* I and S indicate identity and similarity, respectively. E values were obtained by using the BLASTX algorithm and screening the NCBI database.
