# Supplementary material for: The role of horizontal transfer in the evolution of a highly variable lipopolysaccharide biosynthesis locus in xanthomonads that infect rice, citrus and crucifers
Source: BMC Evol Biol. 2007 Dec 6;7:243. doi: 10.1186/1471-2148-7-243 (PMC2238763; doi:10.1186/1471-2148-7-243)
Supplement: Additional file 2 — List of primers used in this study. [file 1471-2148-7-243-S2.doc]

**Additional file 2. List of primers used in this study**

| Primers used for Long Range PCR | |
| --- | --- |
| BPMR1 | TCGGTGACATTGGGCAGTTC |
| BPMR2 | GAATTCGGACATTCTCTACGAGT |
| **Primers used for filling gaps in the shotgun sequence assembly and to confirm the order of genes in the BXO8 LPS locus** | |
| LA1 | ACAAGGCCCTTATCGGTAAACA |
| LA2 | CTAATGTCGAAGCTGCGCAATC |
| LA3 | GAATGGATCGCCCTCCTCACGA |
| LA4 | ATTGGCGCTGAGCACTTACC |
| LA5 | ACGCCGGTGGCCTATGCCCAG |
| LA6 | CTGGGCATAGGCCACCGGCGT |
| LA7 | GCTGCTTGCCTTGCCCTTGCT |
| LA8 | AGATACCTGGCACGACGATCT |
| LA9 | AGATCGTCGTGCCAGGTATCT |
| LA10 | TAGGAACGCCAAAGGAATTAG |
| LA11 | CTGCGCCCCCTGACAACGCTA |
| LA12 | CGATTGAGAAGAAAAGCAGTG |
| LA13 | AGCCGGGCGGAAGGGTCATC |
| LA14 | CTGTGGGCAAAGCCGGACCAGA |
| LA15 | CGCGGTTGCGGGCGGCAAGA |
| LA16 | AGCGAACACGATCGGGTCATCAT |
| LA17 | CTATATGGCCGATACAGCGTA |
| LA18 | TCAACCACACCGATTCAATA |
| LA19 | GTCGGAAATGCCTTTGGTGT |
| LA20 | CGTGTGGATTACGTCGATATCA |
| LA21 | GCAACCGAGTTCACTACCGA |
| LA21 | GCGTCTCGGCATTGCTGGAGC |
| LA23 | GCTCCAGCAATGCCGAGACGC |
| LA24 | CGTAGCTTGTTTCAGGAACG |
