# Supplementary figures and images for: The role of horizontal transfer in the evolution of a highly variable lipopolysaccharide biosynthesis locus in xanthomonads that infect rice, citrus and crucifers
Source: BMC Evol Biol. 2007 Dec 6;7:243. doi: 10.1186/1471-2148-7-243 (PMC2238763; doi:10.1186/1471-2148-7-243)

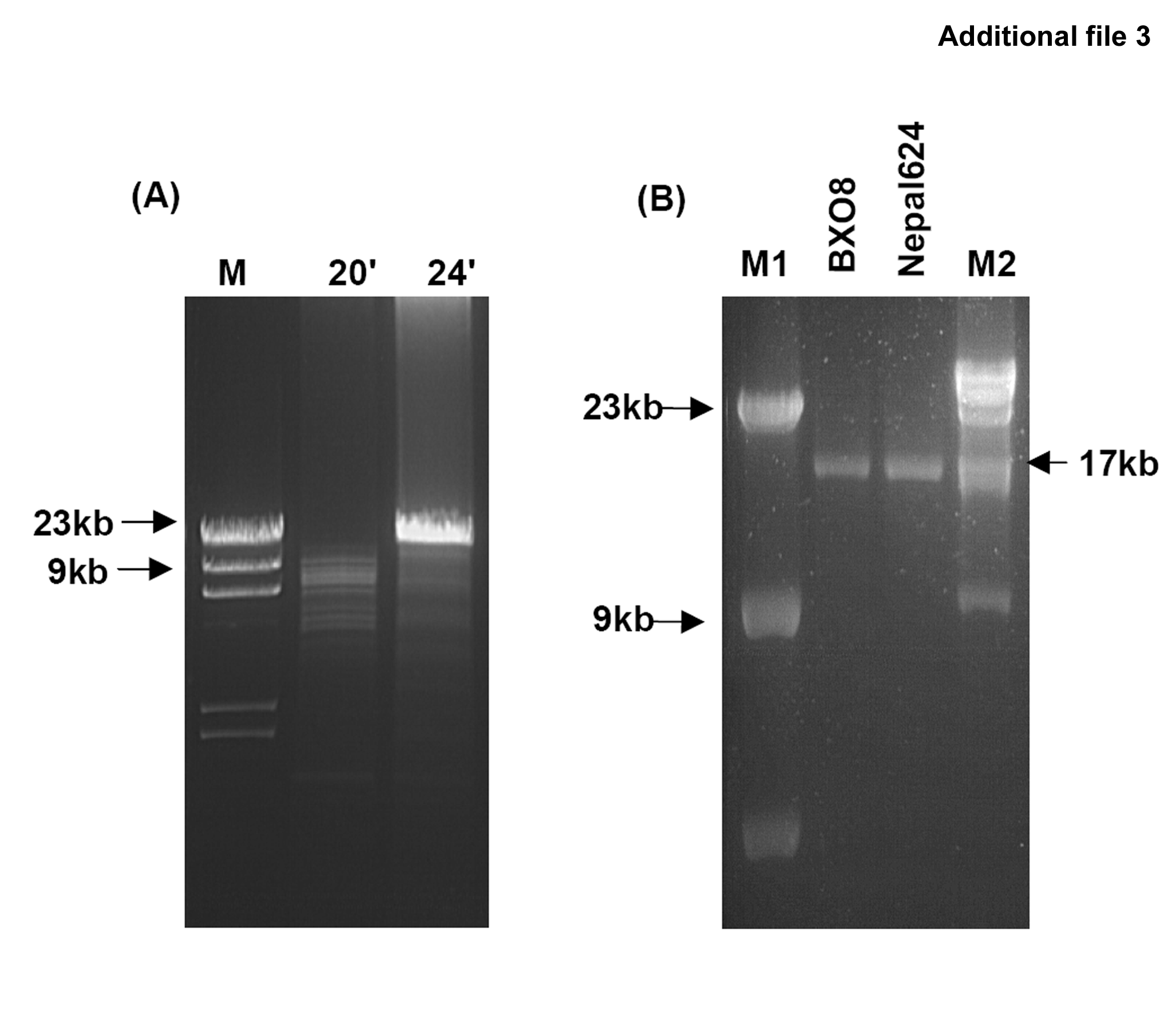

Supplement: Additional file 3 — Amplification of the BXO8 LPS gene cluster using long range PCR. (A) The results of long range PCR using different extension times. A high molecular weight PCR product can be seen only after using an extension time of 24 minutes (see methods). M is the lamda HindIII marker. (B) The size of the PCR product was estimated in both BXO8 (lane2) and Nepal624 (lane3) to be around 18 kb. M1 and M2 are lambda HindIII and lamda monocut mix markers, respectively. [file 1471-2148-7-243-S3.tiff]
