# Supplementary material for: The role of horizontal transfer in the evolution of a highly variable lipopolysaccharide biosynthesis locus in xanthomonads that infect rice, citrus and crucifers
Source: BMC Evol Biol. 2007 Dec 6;7:243. doi: 10.1186/1471-2148-7-243 (PMC2238763; doi:10.1186/1471-2148-7-243)
Supplement: Additional file 4 — List of primers used for resequencing of the BXO8 LPS locus. [file 1471-2148-7-243-S4.doc]

| Additional file 4. List of primers used for resequencing of the BXO8 LPS locus | | | |
| --- | --- | --- | --- |
| Name | Sequence | Primer location on 20070 bp sequence  **(DQ907230)** | Primerdirection |
| RP1 | ATTTCGCCAACTGCTGAGTCGT | 93 – 114 | **🡪** |
| RP2 | ATCTCGCCCTGAGAAATCAGGGTT | 1176 - 1199 | 🡨 |
| RP3 | GTGCGCTTGACGGTGTAAATCT | 997 - 1018 | **🡪** |
| RP4 | CGAAGCATCAGGCAAAAATTGACC | 2188 - 2211 | 🡨 |
| RP5 | CGCAGCGCATTTACTGAAGAGC | 2109 - 2130 | **🡪** |
| RP6 | CAGCACAGACATCGCGACCTTC | 3176 - 3197 | 🡨 |
| RP7 | TTCCAAGATGTTGGGCAACAGC | 3112 - 3133 | **🡪** |
| RP8 | CATGATGCGCATAGGGCTTGGAT | 4182 - 4204 | 🡨 |
| RP9 | CCGCTGCTCATGTTCCAAAAGT | 4112 - 4133 | **🡪** |
| RP10 | AGCCTTTTACCTTGCCAGGTGT | 5251 - 5272 | 🡨 |
| RP11 | GAATCGCTGCCCGTCTGGA | 5161 - 5179 | **🡪** |
| RP12 | AGGCGACACCATCATCGACA | 6085 - 6104 | 🡨 |
| RP13 | CGCTGGGTAAGCTGAGCATTGATT | 6025 - 6048 | **🡪** |
| RP14 | GACGTGGAGCGTGCGATCT | 7099 - 7117 | 🡨 |
| RP15 | TTGTCCGTGCGGATCACCTTC | 7023 - 7043 | **🡪** |
| RP16 | TCGTTTGATCCAGCGCCATT | 8101 - 8120 | 🡨 |
| RP17 | ATCGTGGACTTGGGGTATCGCA | 8016 - 8037 | **🡪** |
| RP18 | AGTTGGCCCGCATTCGCTT | 9079 - 9097 | 🡨 |
| RP19 | CATCACGCAAGCGATGACCA | 9012 - 9031 | **🡪** |
| RP20 | AAAATGACCTCTGTTTCGCCCACC | 10082 - 10105 | 🡨 |
| RP21 | CCAATCAAAATAACAGGTTAGCGT | 10027 - 10045 | **🡪** |
| RP22 | TTCTGTTCTGGCAGCCCAAT | 11126 - 11145 | 🡨 |
| RP23 | TATTGTGTCGCTGAGCCCAGA | 11015 - 11035 | **🡪** |
| RP24 | TGCAAAACTGGTCGGTGCAGA | 12113 - 12134 | 🡨 |
| RP25 | AACCTTCCTGGGTCTGATCACGTA | 11986 - 12009 | **🡪** |
| RP26 | ACTCTCCCGGCGCAATCTATTCAA | 13180 - 13203 | 🡨 |
| RP27 | TTGCCGGCTTCGGAAACCACTT | 13051 - 13072 | **🡪** |
| RP28 | TTTTGTCGCTGCATCGTGCAGT | 14124 - 14145 | 🡨 |
| RP29 | CGCGCTTGATGGTACGAATGAT | 14034 - 14055 | **🡪** |
| RP30 | ATTCCTAGCGCAAGCGTAGATG | 15129 - 15150 | 🡨 |
| RP31 | TTTCCCGCAGCGTTTGCAATAC | 15060 - 15081 | **🡪** |
| RP32 | AAGCAACCGCCCGACGCTAT | 16128 - 16147 | 🡨 |
| RP33 | TCGTAAGGTCTGCCGCAATTGT | 16052 - 16073 | **🡪** |
| RP34 | TGGCAAGAACCACCATCGCA | 17131 - 17150 | 🡨 |
| RP35 | GGCATCATTTGGCATGCACACT | 17048 - 17069 | **🡪** |
| RP36 | TGACGCCTGACCGTCAGCAT | 18163 - 18182 | 🡨 |
| RP37 | GCAAGACCAAGGTGAATTCACTGT | 18030 - 18053 | **🡪** |
| RP38 | TTGGCACGACCGAATTGGCTAT | 19136 - 19157 | 🡨 |
| RP39 | CGTACCAAAGCGTGACCAGT | 19050 - 19069 | **🡪** |
| RP40 | ACGGGAACGGAACGTTTTCG | 19828 - 19847 | 🡨 |
